# Selection of growth-related genes and dominant genotypes in transgenic Yellow River carp *Cyprinus carpio* L.

**DOI:** 10.1007/s10142-018-0597-9

**Published:** 2018-04-05

**Authors:** Lifei Luo, Rong Huang, Aidi Zhang, Cheng Yang, Liangming Chen, Denghui Zhu, Yongming Li, Libo He, Lanjie Liao, Zuoyan Zhu, Yaping Wang

**Affiliations:** 1 State Key Laboratory of Freshwater Ecology and Biotechnology, Institute of Hydrobiology, Chinese Academy of Sciences, Wuhan, 430072 China; 2 University of Chinese Academy of Sciences, Beijing, 100049 China

**Keywords:** Transgenic Yellow River carp, Growth-related genes, Genotype, Bulked segregant analysis (BSA), Genome-wide association study (GWAS)

## Abstract

Transgenic Yellow River carp is characterized by rapid growth rate and high feed-conversion efficiency and exhibits a great application prospect. However, there is still a significant separation of growth traits in the transgenic Yellow River carp family; as such, growth-related genotypes must be screened for molecular marker-assisted selection. In this study, 23 growth-related candidate genes containing 48 SNP markers were screened through bulked segregant analysis (BSA) among transgenic Yellow River carp family members showing significant separation of growth traits. Then, two growth-related genes (Nos. 17 and 14 genes) were identified through combined genome-wide association study (GWAS) of candidate genes and validation of the full-sibling family approach. Nos. 17 and 14 genes encode BR serine/threonine-protein kinase 2 (BRSK2) and eukaryotic translation-initiation factor 2-alpha kinase 3 (Eif2ak3), respectively. The average body weight of three subgroups carrying the genotypes 17GG, 17GG + 14CC, and 17GG + 14TT of these two genes increased by 27.96, 38.28, and 33.72%, respectively, compared with the controls. The proportion of individuals with body weight > 500 g in these subgroups increased by 19.22, 26.82, and 30.92%, respectively. The results showed that appropriate genotype carriers can be selected from the progeny population through BSA sequencing combined with simplified GWAS analysis. Hence, basic population for breeding can be constructed and transgenic Yellow River carp strains with stable production performance and uniform phenotypic properties can be bred.

**Electronic supplementary material:**

The online version of this article (10.1007/s10142-018-0597-9) contains supplementary material, which is available to authorized users.

## Introduction

Since the 1980s, transgenic technology has gained increasing attention for genetic improvement of farmed fish; scholars in the USA, Canada, Britain, Norway, Japan, and South Korea have carried out transgenic fish breeding research on almost all important farmed fish for traits such as growth, nutrition, and disease resistance (Zhu et al. 1985; Maclean and Laight 2000). The growth rate and feed conversion efficiency of transgenic fish have significantly improved, and they show tremendous industrialization prospects (Cook et al. 2000; Nam et al. 2001). In November 2015, the US Food and Drug Administration approved the launch of the fast-growing genetically modified Atlantic salmon, which became the world’s first genetically modified animal food allowed in the market; this year marks the era of transgenic fish industrialization (Ledford 2015).

Zhu et al. from the Institute of Hydrobiology, Chinese Academy of Sciences, successfully developed the fast-growing growth hormone (GH) gene transgenic carp through microinjection of the fertilized eggs of Yellow River carp (Zhu et al. 1986; Zhu et al. 1989; Zhu et al. 1990; Wang et al. 2001a, b). The exogenous GH gene was continuously expressed at high levels in the transgenic carp, which exhibited growth rate twice higher than that of the control and 16.8% increase in the feed conversion efficiency. Non-transgenic carp need 2 years to achieve the listed specifications, whereas transgenic carp only need 1 year before being available in market and thus display great industrial prospect (Fu et al. 2007; Duan et al. 2009).

Study of fast-growing transgenic carp showed that although their offspring carried the same integration site (Chen et al. 2015), their growth traits significantly varied and the frequency distribution of their body weight and length presented two obvious peaks (unpublished data). We speculate that growth traits are mainly associated with the genotypes of specific genes in the genome of the receptor (fertilized eggs undergoing microinjection). Therefore, breeding of transgenic Yellow River carp requires the coordination of specific genotypes in the receptor genome. In this regard, we must first explore genes related to growth traits in the genome of transgenic carp.

Genotype/phenotype correlation analysis has become an efficient method for functional genomics research. Genome-wide association study (GWAS) was first used to identify age-related macular degeneration-related genes in the human genome (Klein et al. 2005). In 2015, Ayllon et al. found 76 SNPs in the 230-KB region related to sexual maturity and located on chr25 through GWAS analysis of Atlantic salmon (Ayllon et al. 2015). In research of crops, genes related to tiller number, amylase content, grain length, grain weight, flowering time, and rice production were detected by GWAS (Huang et al. 2010; Huang et al. 2012). With the development of DNA high-throughput sequencing technologies and the gradual maturity of supporting analysis platforms, bulked segregant analysis (BSA) has become popular as a more efficient and faster method than GWAS (Michelmore et al. 1991). Compared with GWAS, BSA is more suitable for locating related genes in populations with significantly separated phenotypic traits. Voz et al. located genes related to ethylnitrosourea-induced pancreatic hypoplasia through BSA (Voz et al. 2012). This method was also used to locate the powdery mildew resistance gene pm5.1 in cucumber (Nie et al. 2015). In the same year, Takagi et al. used BSA to identify the salt-tolerant genes of rice; the genes were then used to cultivate new salt-tolerant rice varieties that could be available within 2 years, which greatly shortened the breeding period (Takagi et al. 2015). Hence, BSA has been successfully used to explore genes related to resistance and economic traits in a variety of animals and plants.

In recent years, research on the fish genome has greatly progressed in China. Whole-genome sequencing was conducted in some important economical fish, such as half-smooth tongue sole (*Cynoglossus semilaevis*) (Chen et al. 2014), common carp (*Cyprinus carpio* L.) (Xu et al. 2014), and grass carp (*Ctenopharyngodon idellus*) (Wang et al. 2015). Comprehensive analysis of the genome sequences of cultured species will provide an important basis for functional genomic research and genetic improvement. Information on whole-genome sequences of common carp will play an important role in the breeding of transgenic carp.

In this study, a pair of transgenic Yellow River carp parents was used to construct a full-sibling family with significantly separated growth traits. Later, growth-related genes and dominant genotypes of the transgenic Yellow River carp were screened through BSA sequencing combined with simplified GWAS analysis. The detected growth-related genes and dominant genotypes were validated in the constructed full-sibling family. Our findings can be used to select individuals with appropriate genotypes for constructing a breeding population, which can be used to breed transgenic Yellow River carp with stable production performance and uniform phenotypic properties.

## Materials and methods

### GH transgenic Yellow River carp and ethical procedures

P_0_ GH transgenic Yellow River carp were produced by microinjection of fertilized eggs with the gene construct pCAgcGH containing the grass carp (*C. idellus*) GH gene (gcGH), whose expression is driven by the β-actin gene promoter of common carp (*C. carpio* L*.*) (Wang et al. 2001a, b). F_1_ transgenic carp were obtained by P_0_ transgenic male (sperm revealed to be pCAgcGH positive) × Wt female crosses. F_2_ fish were obtained by crossing F_1_ transgenic male with Wt female. F_3_ generation was produced by a similar method. The homozygous transgenic fish TG3 line was obtained by intercrossing F_3_ transgenic males with females, which were derived from the same strain that possessed a transgene integrated at the same locus in the genome with the same copy number (Zhong et al. 2012). In the experiment, parents for the construction of the full-sibling family were selected from the TG3 line.

Animal welfare and experimental procedures were carried out in accordance with the Guide for the Care and Use of Laboratory Animals (Ministry of Science and Technology of China, 2006). Ethical approval for the work and field permits for the collection of fish were obtained from Expert Committee of Biomedical Ethics, Institute of Hydrobiology of the Chinese Academy of Sciences. The Reference number obtained was Y119021F01.

### Construction of a full-sibling family and measurement of growth traits

One male (TM) and one female (TF) fish were randomly selected from the 3-year-old TG3 line. Then, one-to-one conventional wet insemination was used to obtain transgenic sibling group fertilized eggs, which were hatched in the mesh. About 5000 1-month-old juveniles were placed in a 600-m^2^ pond for breeding. At the age of 4 months, 442 fish from 5000 individuals were randomly selected. Body weight and length were measured for plotting of frequency distribution map. Based on the peak distribution in the map, the full-sibling group was divided into fast- and slow-growing subgroups.

### BSA analysis of offspring

Thirty individuals were selected from the nearest peak region in fast- and slow-growing subgroups. DNA was extracted from the tail fin by using the phenol–chloroform method. DNA pools were constructed by mixing equal DNA, hereinafter referred to as fast and slow DNA pools. The two DNA pools were used to construct two libraries by using the NEBNext Ultra DNA Library Prep Kit for Illumina (NEB, USA) according to the manufacturer’s instructions. The libraries were run in three lanes on Illumina HiSeq 2000 by using 125 base paired-end reads (v3 chemistry kit, Illumina, USA), and the sequencing fragments were 300–400 bp long.

BSA analysis was performed by mapping the clean data of the fast and slow DNA pools to the carp reference genome (V2.0.Commoncarp.gfasta, http://www.carpbase.org/) by using the BWA software mem algorithm with default parameters. Samtools was used for SNP calling and annotation of the two pools (the number of supported reads for each allele was not less than 4, and the quality of the SNP was not less than 20). The difference in allele frequencies was calculated using the population2 software and expressed as Fst value. The Fst value was calculated as follows:Fst = (Pi_total−Pi_within)/Pi_totalPi_within = (Pi_population1 + Pi_population2)/2Pi:1−fA^2^–fT^2^–fC^2^–fG^2^

Fst calculation was performed for each SNP. For one SNP locus, fN denotes the frequency of nucleotide N (A T C G) in the SNP locus from one group, and Pi_within represents the average Pi value of the two groups considered in the study. Pi_total represents the total Pi of the allele frequencies of the two groups. SNP loci of Fst > 0.5 and coverage > 20× were selected. SNP position information was annotated with the Perl program, and gene function was predicted using the blast2go software to determine candidate genes. Genes with SNPs located in the promoter or exon regions were selected as candidate genes.

### Screening, amplification, and re-sequencing of SNP loci in candidate genes

From each candidate gene, we selected two adjacent SNP loci (one was heterozygous and the other one was homozygous in another parent) to detect the genotype of each individual by re-sequencing. Primers were designed for both sides of the selected SNP site for each candidate gene (Additional file 1: Table S1). F- and R-barcode sequences were added to the 5′ end of upstream and downstream primers, respectively, to distinguish different individuals. The barcode sequences are listed in Additional file 2: Table S2. The corresponding barcode combination for each individual was provided in Additional file 3: Table S3. The length of the PCR product was controlled within 290 bp.

The DNA of 442 individuals from the full-sibling family was extracted using the Universal Genomic DNA Kit (CWBIO, China). The selected SNP sites of these individuals were amplified with the corresponding F- and R-barcode primers. An equivalent amount of PCR products was blended, and the mixed sample was purified with the Gel Extraction Kit (OMEGA, USA). A sequencing library was then constructed with the purified product by using the NEBNext Ultra DNA Library Prep Kit for Illumina (NEB, USA) according to the manufacturer’s protocol. The read length was PE150, and R1- and R2-read data were generated.

### Genotype/phenotype association analysis of candidate genes

The 9th to 20th bases of all F- and R-barcode fragments were captured and completely mapped with R1- and R2-read by using the BWA software. The read pairs that could match both F and R barcodes were exported. According to the information of the F- and R-barcode sequences of the individuals, the read pairs were split into 442 samples.

The read pairs of the 442 samples were aligned using BWA (default parameters were used as alignment parameters) with SNP amplification regions as reference sequences and indexed with Samtools. SNPs were identified by GATK 3.4. SNPs with minor allele frequency > 0.05 and call rate > 90% were screened. The genetic relationship between the genotype data that met the requirements and the phenotype data was calculated using Tassel5.0, with the parameter of the Kinship method = Centered_IBS, Max Alleles = 6, and genotype data imported using the hapmap format. The association between the genotype and phenotype data was analyzed using the MLM model, with the parameter setting as Compression Level = Optimum Level and Variance Component Estimation = P3D (estimate once). A Manhattan plot was generated using the Manhattan Plot function in the Tassel 5.0 software.

### Verification of growth-related genotypes and screening of the combinations of growth-associated genotypes

According to the correlation analysis results, SNP sites with *p* < 0.1 were selected and amplified in the 442 individuals of the full-sibling family by single PCR reactions. The PCR products for all individuals were sequenced by the Sanger method for genotyping. Genotype data were analyzed together with the corresponding body weight and length data. Firstly, SNP loci with *p* < 0.05 were analyzed at a single SNP site level, and the phenotype data of each genotype were evaluated using one-way analysis of variance (ANOVA) with SPSS19.0. SNP locus genotypes with significantly different growth traits (*p* < 0.05) were obtained. In addition, some single SNP may not be significantly correlated considering that the growth traits are quantitative traits. Therefore, the *p* value was amplified to *p* < 0.1 in the genotype combination analysis. Manual analysis of the two combinations of genotypes in different SNP loci was then performed. Meanwhile, the average weight and the proportion of individuals with body weight > 500 g for each genotype or genotype combination was counted. As a result, we could obtain advantage genotypes or genotype combinations of growth traits.

## Results

### Analysis of growth traits in the full-sibling family

**Fig. 1:**
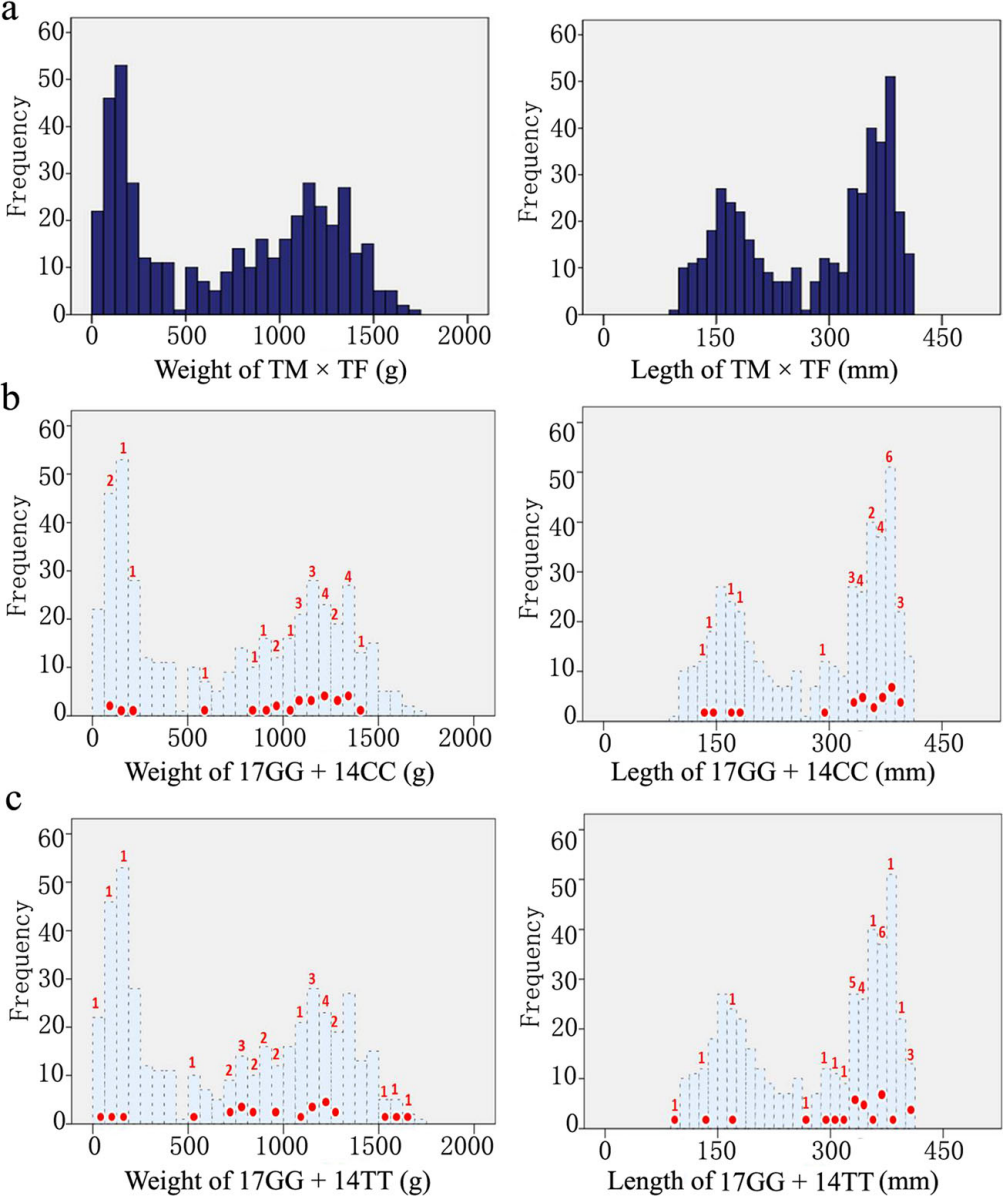
Frequency distribution of body weight and length. **a** Frequency distribution of body weight and length for 442 offspring in the transgenic full-sibling family (TM × TF). The frequency distribution map was created using SPSS19.0. A significant separation was detected between the two peaks for body weight and length. **b** Frequency distribution of a subgroup (17GG + 14CC) was dominant in the frequency map of the full-sibling family. **c** Frequency distribution of a subgroup (17GG + 14TT) was dominant in the frequency map of the full-sibling family. Black dashed line of light blue filled area represents the frequency distribution of the weight or body length of the full-sibling family, as the background. The red dots indicate the range in which the genotype combination individuals are distributed, and the red numbers represent the numbers corresponding to the red dots

A transgenic full-sibling family was obtained by mating of TM and TF from the transgenic TG3 line. The body weight and length of 442 individuals in the full-sibling family were measured at the age of 4 months. The frequency distribution of body weight and length of the 442 individuals did not comply with the normal distribution (Fig. 1a). A significant separation was detected between two peaks in the frequency distribution of body weight and length. For the fast-growing subgroup, the body length range was 280.00–410.00 mm, the body weight range was 530.00–1726.00 g, the average body length was 356.16 mm, and average body weight was 1120.10 g. For the slow-growing subgroup, the body length range was 93.00–279.00 mm, the body weight range was 27.00–528.00 g, and the average body length and weight were 174.67 mm and 186.74 g, respectively. In addition, the average body length and weight of the full-sibling family were 278.14 mm and 718.88 g, respectively, and the number of individuals with body weight > 500 g was 258 (accounted for 58.37% of the total).

### Growth-related candidate genes identified by offspring BSA

According to the BSA classification principle, 30 individuals were selected in the nearest peak area from the fast- and slow-growing subgroups to construct fast and slow DNA pools. About 692,329,016 and 869,508,434 clean reads and 86.54 and 91.03 G clean data were obtained by re-sequencing of the fast and slow DNA pools, respectively. The clean data of the fast and slow DNA pools were mapped to the reference genome of the common carp, with mapped rates of 94.29 and 94.13%, average depths of 47.48× and 37.46×, and 1× coverage values of 98.95 and 99.32%, respectively. This finding confirmed the high quality of the sequencing data and their suitability for further mutation detection and correlation analysis.

**Fig. 2:**
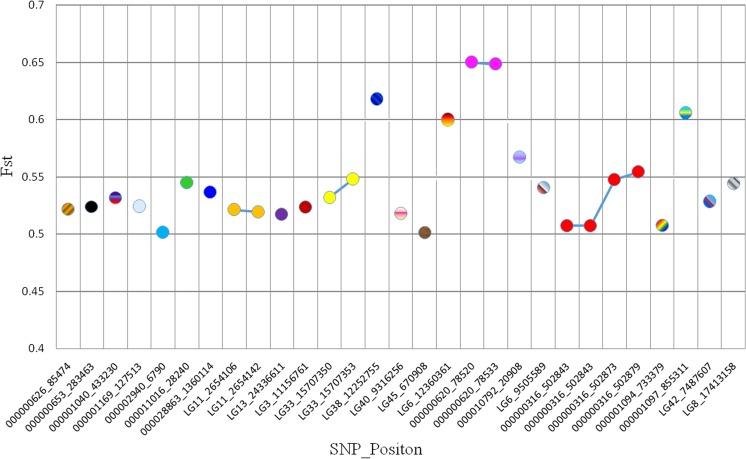
29 SNP loci anchored in the regions of 23 genes. The horizontal coordinate represented the 29 SNP loci with Fst > 0.5. The vertical coordinate represented Fst values of SNPs. SNPs with the same color, which were linked together, were located on the same gene

A total of 18,304,028 and 17,879,410 SNP sites were detected from the fast and slow DNA pools and annotated using Samtools. The Fst value representing the frequency difference of SNPs in the two pools was calculated, and 275 SNPs with Fst > 0.5 and coverage > 20× were obtained. According to the annotated results, 29 SNPs (Fig. 2) were distributed in the exon or promoter regions of 23 genes (Table 1), which were used as candidate genes for subsequent studies. The gene functional annotation results showed that most of these genes, such as Eif2ak3 and Pleiotrophin, play a role in transcriptional and metabolic pathways.

**Table 1:** Candidate genes screened by BSA and annotated by gene ontology

Order number	SNP position	Gene ID	Gene ontology
1	000000626_85474	CAFS_CommonC_T_00025105	zg57_xenla
2	000000653_283463	CAFS_CommonC_T_00025570	cypCar_00045517, partial
3	LG45_670908	CAFS_CommonC_T_00228282	pccb_bovin
4	000010792_20908	CAFS_CommonC_T_00089016	dpp10_human
5	000002940_6790	CAFS_CommonC_T_00074102	frim_salsa
6	000011016_28240	CAFS_CommonC_T_00089291	med9_mouse
7	000028863_1360114	CAFS_CommonC_T_00099446	tprn_rat
8	LG11_2654106 LG11_2654142	CAFS_CommonC_T_00128778	gtpb2_mouse
9	LG13_24336611	CAFS_CommonC_T_00134509	pcyxl_mouse
10	LG3_11156761	CAFS_CommonC_T_00177972	s22a3_mouse
11	LG33_15707350 LG33_15707353	CAFS_CommonC_T_00192291	pwp2b_human
12	LG38_12252755	CAFS_CommonC_T_00206348	herc1_human
13	LG40_9316256	CAFS_CommonC_T_00216313	60s ribosomal protein l7-like 1
14	000001040_433230	CAFS_CommonC_T_00041901	eif2ak3_rat
15	LG6_12360361	CAFS_CommonC_T_00246152	ets1b_xenla
16	000000620_78520 000000620_78533	CAFS_CommonC_T_00024951	cmp-n-acetylneuraminate-beta-galactosamide-alpha- -sialyltransferase 1
17	000001169_127,513	CAFS_CommonC_T_00047389	brsk2_mouse
18	LG6_9505589	CAFS_CommonC_T_00245847	cel2a_rat
19	000000316_502843 000000316_502843 000000316_502873 000000316_502879	CAFS_CommonC_T_00012481	t4s1_mouse
20	000001094_733379	CAFS_CommonC_T_00043863	fakd3_bovin
21	000001097_855311	CAFS_CommonC_T_00044007	kld10_human
22	LG42_7487607	CAFS_CommonC_T_00220443	loxh1_mouse
23	LG8_17413158	CAFS_CommonC_T_00250347	pleiotrophin isoform ×3

### Genotype/phenotype correlation analysis of SNP loci in candidate genes

**Fig. 3:**
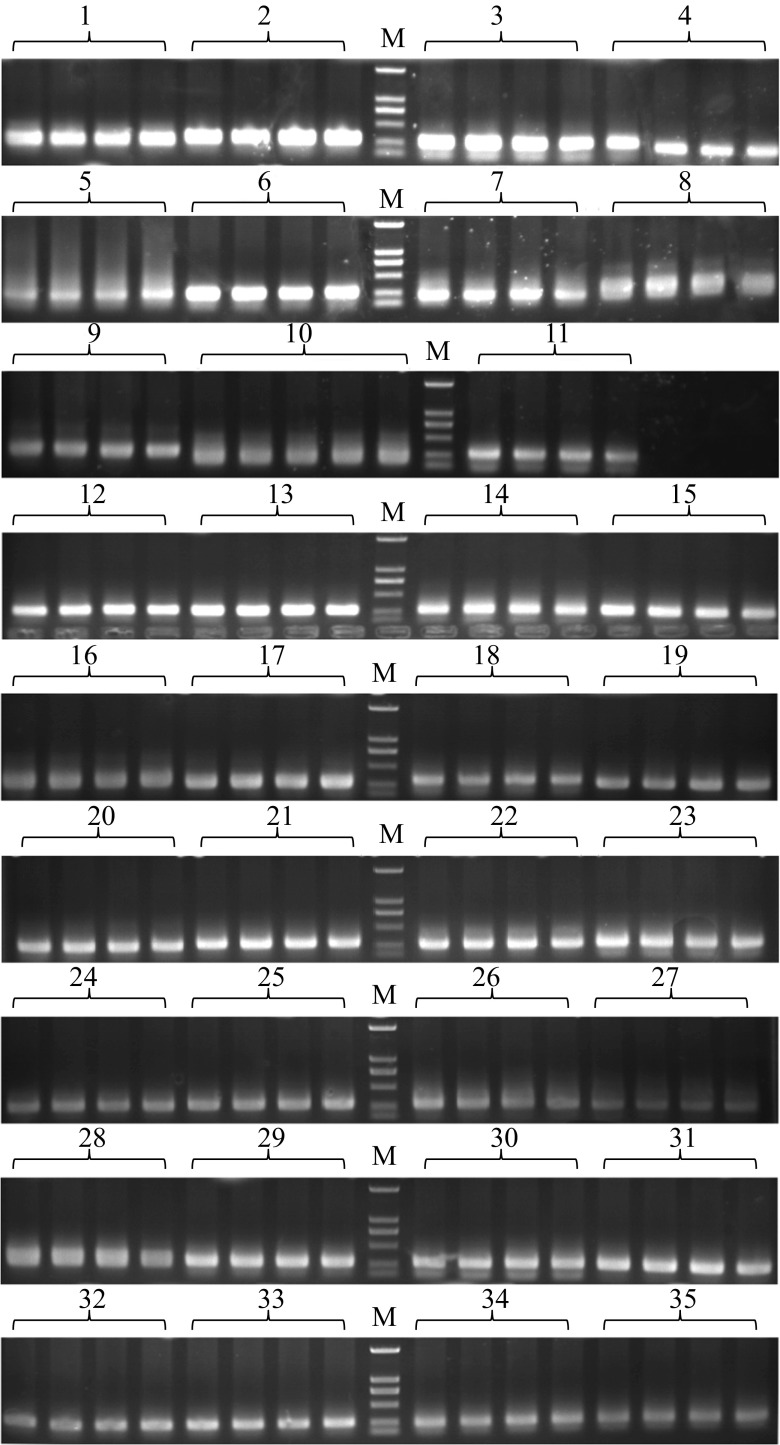
Gel picture of 35 mixed samples. 1–35 represent 35 amplification products in 442 mixed samples, M: DS2000 marker

**Fig. 4:**
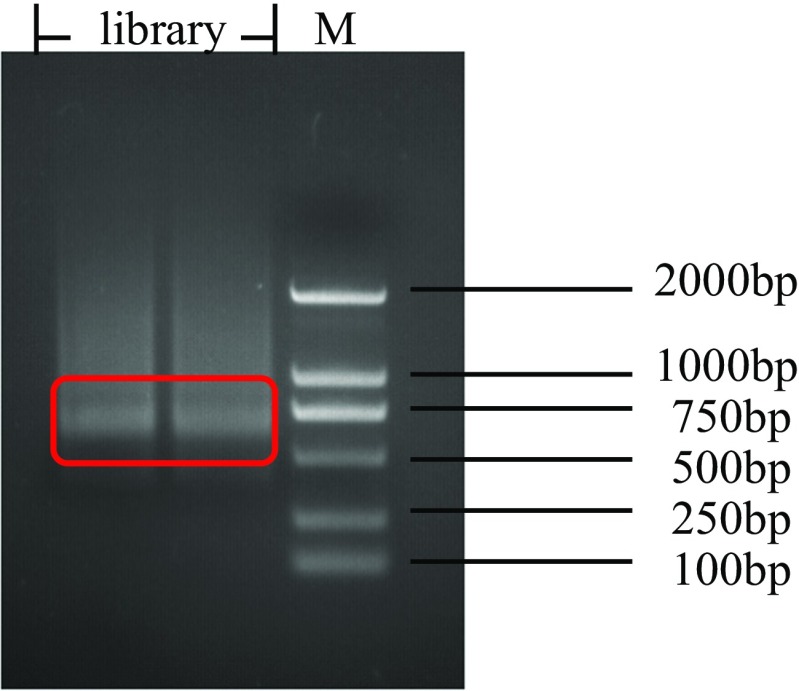
Gel picture of 48 SNP locus library. The length of the library is within 400–600 bp, which is shown in red square. M: DS2000 marker

**Fig. 5:**
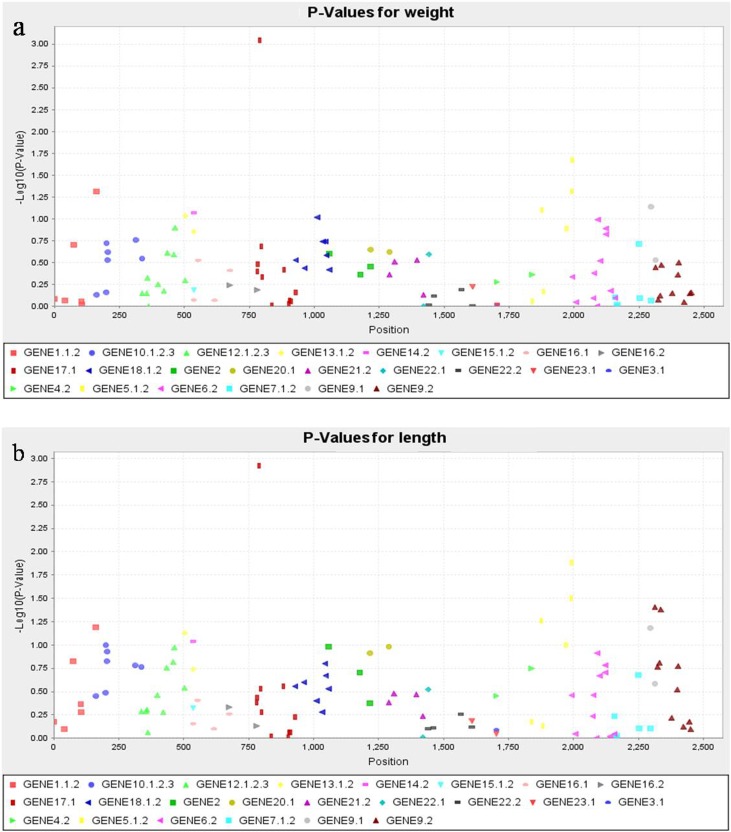
Manhattan plot for the correlation analysis of body weight and length. **a** Manhattan plot for the correlation analysis of body weight. **b** Manhattan plot for the correlation analysis of body length. The horizontal coordinate represents the SNP locus, and the vertical coordinate represents the –log10 (*p* value). The –log10 (*p* value) was plotted for each SNP in chromosomal order. The spacing between SNPs on the plot is uniform and does not reflect the distances between SNPs on the chromosomes

A total of 48 SNP loci from 23 candidate genes were selected to genotype 442 individuals. First, 35 pairs of specific primers (Additional file 1: Table S1) were designed for 48 SNP loci. For each pair of primers, 5 μl of the amplification products in 442 individuals (for example, the amplifications of locus 23 are shown in Additional file 4: Fig. S1) were respectively blended into one sample. About 150–200 μl of the product was collected for electrophoresis (Fig. 3) and purification. After measuring the concentration, equal amounts of 35 DNA samples were blended into one sample and used for library construction. The gel picture of the library is shown in Fig. 4. After sequencing, the sequencing data of R1- and R2-read, including 39,145,182 clean reads and 5.54 G of clean data, were obtained. According to the F- and R-barcode sequences at both ends of each individual, the clean data of R1- and R2-read were successfully split into 442 samples. The read pairs of the 442 samples were compared with the reference sequence to obtain the genotype data for each sample. A correlation analysis was then performed based on the genotype and phenotype data of 442 individuals. The results of the genotype/phenotype correlation analysis using the MLM model are presented in Additional file 5: Table S4. Small *p* values indicate strong correlation. The genetic marker effect values are shown in Fig. 5; the -log10^*P*^ values of all SNP sites obtained using the *F* test were sorted by gene order. Small *p* values indicate a large vertical coordinate. Six statistically significant SNP loci (*p* < 0.05) corresponding to four genes (Nos. 17, 5, 9, and 1) were obtained. In addition, the genotype combination analysis with the range of *p* values amplified to *p* < 0.1 showed that the associated SNP loci increased to 12, which correspond to seven genes (Nos. 13, 14, and 18 in addition to the four mentioned genes).

### Growth trait-related genotypes and genotype combinations identified by verification in the full-sibling family

**Fig. 6:**
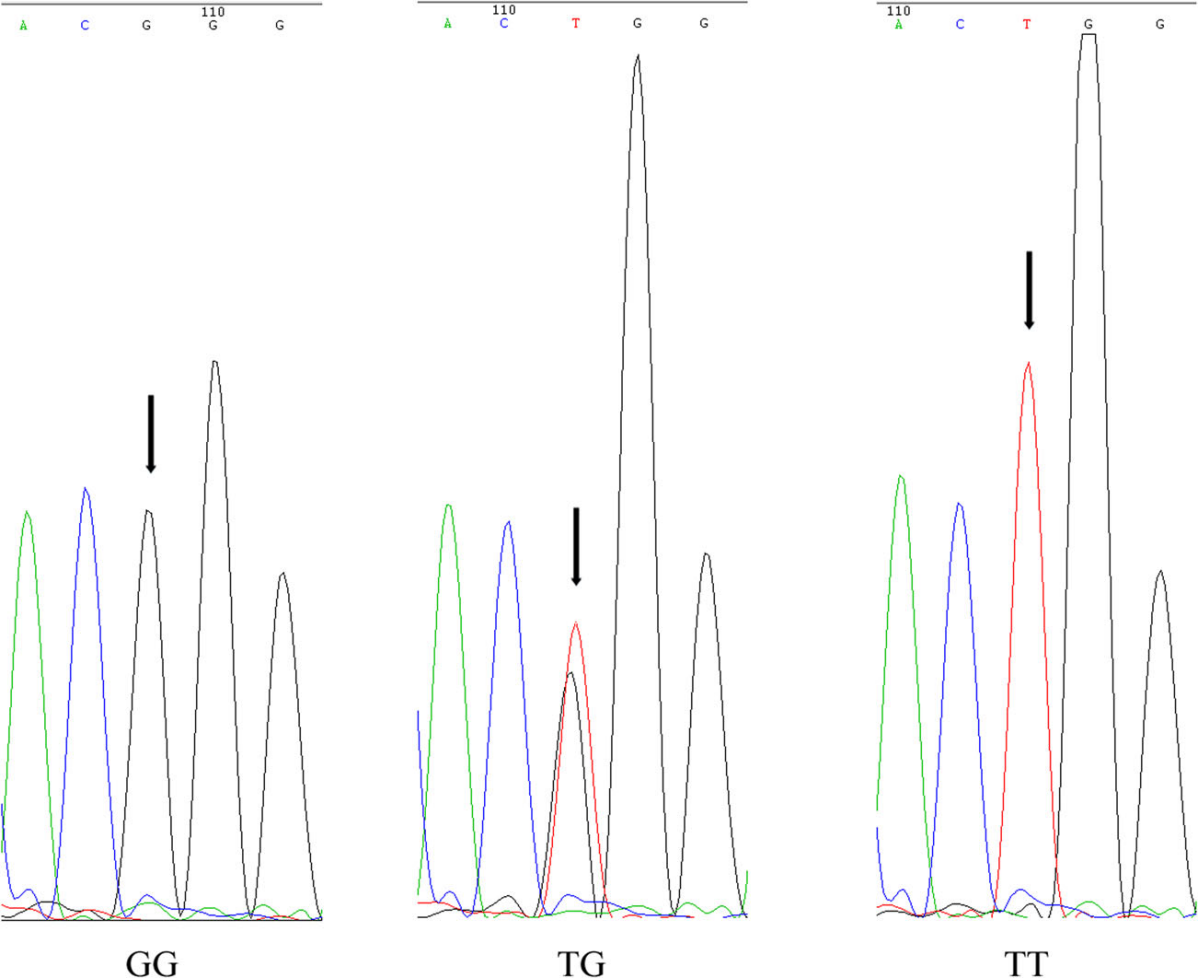
DNA sequencing map of the G + 127,513T site in the No. 17 gene. SNP site is indicated by black arrow

After correlation analysis, seven candidate genes were obtained and verified in the full-sibling family by single PCR reactions. The amplified SNPs of the seven genes in 442 individuals were sequenced by the Sanger method, aligned with the DNAMAN6.0 software, and analyzed with Chromas to obtain the corresponding genotypes. The results of one-way ANOVA showed significant differences in body length and weight among the three subgroups carrying the genotypes GG, TG, and TT (Fig. 6) at the G + 127,513T locus of the No. 17 gene (*p* < 0.01). The average lengths of the individuals with GG, TG, and TT genotypes were 316.34, 283.19, and 221.83 mm, respectively, and the average weights were 919.87, 746.91, and 419.12 g, respectively (Table 2). When compared with the TG and TT individuals, the GG individuals exhibited obvious advantages with respect to body length and weight. In the GG genotype subgroup, the proportion of individuals with body weight > 500 g accounted for 77.59%, which is approximately 20% higher than the 58.37% of the full-sibling family. GG was found to be the dominant genotype.

**Table 2:** Statistical data for body length and weight of subpopulations carrying different genotypes and the full-sibling family

Genotype	Number	Number (body weight > 500 g)	Frequency (body weight > 500 g) (%)	Body length (mm)	Body weight (g)
Full-sibling family	442	258	58.37	278.14 ± 96.80B	718.88 ± 512.59B^*^
17GG	116	90	77.59	316.34 ± 84.13A	919.87 ± 461.77A^*^
17TG	227	138	60.79	283.19 ± 95.39B	746.91 ± 510.23B^*^
17TT	99	30	30.30	221.83 ± 88.82C	419.12 ± 437.46C^*^
17GG + 14CC	27	23	85.19	331.81 ± 77.83	994.04 ± 409.23
17GG + 14CT	61	42	68.85	303.77 ± 89.42	868.03 ± 503.97
17GG + 14TT	28	25	89.29	328.79 ± 76.08	961.29 ± 411.31
17TG + 14CC	67	42	62.69	282.88 ± 89.72	728.04 ± 486.65
17TG + 14CT	110	67	60.91	282.48 ± 98.56	746.30 ± 515.64
17TG + 14TT	50	29	58.00	285.16 ± 97.55	773.52 ± 537.67
17TT + 14CC	23	7	30.43	233.78 ± 94.42	490.26 ± 508.70
17TT + 14CT	52	18	34.62	230.31 ± 89.88	447.75 ± 450.66
17TT + 14TT	24	5	20.83	192.00 ± 77.05	288.92 ± 304.86

^*^Different big letters in the same column indicate significant difference at *p* < 0.01

**Fig. 7:**
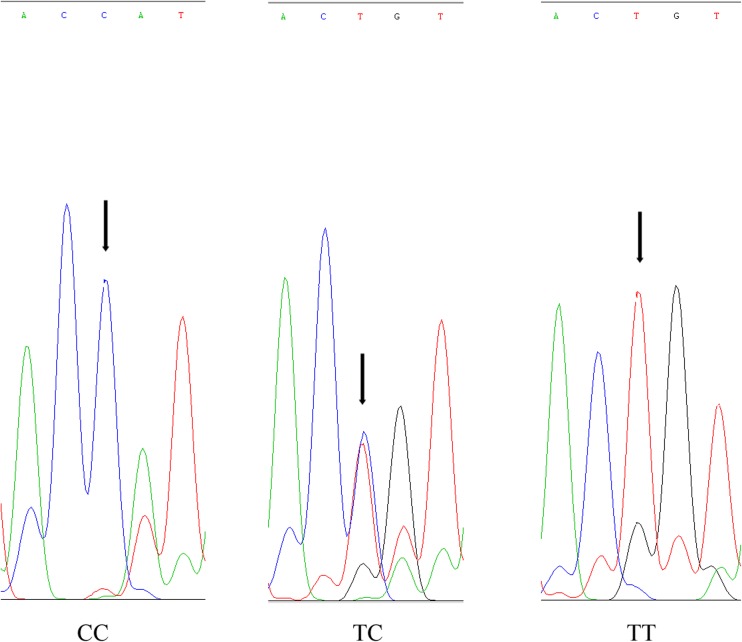
DNA sequencing map of the C + 433,227T site in the No. 14 gene. SNP site is indicated by black arrow

The two combinations of genotypes in different SNP loci were manually analyzed. The results showed that the subgroups carrying the genotype GG of the G + 127,513T site in the No. 17 gene combined with the genotypes CC and TT (Fig. 7) of the C + 433,227T site in the No. 14 gene (17GG + 14CC and 17GG + 14TT) exhibited obvious advantages in terms of growth traits; for these subgroups, the average lengths were 331.81 and 328.79 mm, and the average weights were 994.04 and 961.29 g. The frequency distributions of the body weight and length of these two subgroups were dominantly shown in the frequency map of the full-sibling family (Fig. 1b, c). Most individuals were enriched in the peak area and showed high body weight or length. The proportion of these two genotype individuals with body weight > 500 g accounted for 85.19 and 89.29%, which are approximately 30% higher than the 58.37% of the full-sibling family. The advantages were very evident. Considering the limited number of subgroups (27 and 28, respectively), we did not perform one-way ANOVA.

## Discussion

Traditional GWAS comprises genome-wide re-sequencing of each individual in a natural population with rich genetic diversity and can be conducted by combination of certain statistical methods. This strategy can be used to localize multiple target trait genes but is time consuming and costly. The principle of BSA is to construct two DNA mixing pools from two groups with relative traits and identify different genomic regions of the two mixed pools. Loci related to traits are then detected and annotated. BSA can quickly locate trait loci at a low cost. In this study, based on the characterization of the separation in the growth traits of transgenic Yellow River carp, we used Pool-Seq technology and effectively combined BSA and GWAS to explore trait-related genes. This technique not only improved the detection efficiency of trait-related genes but also reduced the cost of sequencing. The improvement in detection efficiency was reflected in that BSA was performed to focus on trait-related genes among dozens of candidate genes (in this study, we focused on 23). GWAS was then conducted on these candidate genes to obtain a small number of effective trait-related genes (4–7 genes were found in this study). Cost reduction was mainly reflected in the use of the Pool-Seq method: first, mixing of similar samples for sequencing greatly reduced the number of libraries, and second, overall sequencing volume and workload of data analysis were reduced.

However, this method presents certain limitations. For example, when the 35 PCR products of the 48 SNP loci in the 442 individuals were mixed and sequenced, the detection rate of all SNP loci was 77%; the remaining loci with call rate < 90% indicated that the site could not be detected. When the sequencing data were split into 442 samples according to the barcode label, only 67% of the sequencing data were split into 442 samples because of the mixed uniformity of the PCR products and the non-specific amplification of PCR. Schlötterer et al. reported that Pool-Seq requires the mixing of DNA from a large number of samples; as such, different amounts of DNA from individuals in the pool will have a potential negative effect on the results (Schlötterer et al. 2014). Nevertheless, the authors mentioned that the impact of this problem can be easily reduced by increasing the number of individual samples (Schlötterer et al. 2014). The number of samples may vary depending on group size. If the number of groups is limited, then we can try to mix the same amount of samples from the same parts of tissue to extract DNA or perform quantitative analysis by using the Qubit DNA Assay Kit (Life Technologies, USA). In addition, with respect to the problem of non-specific PCR amplification, we need to perform further screening of the designed primers by sequencing the PCR products; this technique can potentially avoid the phenomenon in which the amplification length is the same but the product is actually non-specifically amplified.

In this study, the growth trait-related genes of the common carp were focused on the Nos. 17 and 14 genes, which encode BR serine/threonine-protein kinase (BRSK2) and eukaryotic translation initiation factor 2-alpha kinase 3 (Eif2ak3), respectively. BRSK2 plays a key role in the polarization of neurons and axonogenesis, cell cycle progression, and insulin secretion (Müller et al. 2010; Nie et al. 2012; Chen et al. 2012) and regulates the reorganization of the actin cytoskeleton. BRSK2 may also play a role in the apoptotic response triggered by endoplasmic reticulum (ER) stress (Wang et al. 2012). Eif2ak3, also known as protein kinase R (PKR)-like ER kinase (PERK), phosphorylates and inactivates the alpha subunit of eukaryotic translation-initiation factor 2, leading to rapid reduction of translational initiation and repression of global protein synthesis (Shi et al. 1998; Harding et al. 1999; Hayes et al. 1999). Eif2ak3 is a type I membrane protein located in the ER, where it is induced by ER stress caused by mal-folded proteins (Harding et al. 1999). These two genes are involved in cell cycle progression, protein synthesis, and metabolism; however, the relationship of the roles of these two genes to the growth traits of the common carp needs to be further clarified.

In our experiment, the parents for full-sibling family construction were selected from a transgene homozygous line that carried the same integration site. However, the reason behind the significant separation of growth traits in the full-sibling family remains unknown. Basing on the analysis of the growth traits of three different genotype subgroups of the No. 17 gene and the previous study on feeding traits of transgenic carp (Duan et al. 2009; Duan et al. 2010), we speculate that this phenomenon, on one hand, may be due to the feeding behavior of transgenic carp. Under limited nutritional conditions, competition for feeding occurs, and fish with relatively small body size face severe pressure to survive, leading to the emergence of many small-sized fish (Duan et al. 2009). On the other hand, we believe that this phenomenon is relevant to the genotypes of specific genes in the receptor genome. The possible mechanism is that certain genotypes with phenotypic effects are selectively amplified under the “new normal” state of sustained expression of the transgene, leading to significant phenotypic segregation. When compared with the genotypes of the parents, the genotypes of some progeny in the full-sibling family were recombined, resulting in greater separation of individual growth traits in the family. This phenomenon also provides an important clue for marker-assisted selection breeding of transgenic carp.

The proposed research scheme is suitable for low-cost discovery of trait-related genes in some families with distinct traits. However, the number of samples and uniformity of the mixed PCR products must be further improved.

In conclusion, we found that the major gene No. 17 and the helper gene No. 14 are related to the growth traits of transgenic Yellow River carp. The growth traits of three subgroups with the genotypes 17GG, 17GG + 14CC, and 17GG + 14TT were improved by 20–30%. Our results provide important genetic evidence and basis for marker-assisted selection for breeding of transgenic Yellow River carp.

## Electronic supplementary material

Table S1 Specific primers for each SNP site. (GIF 2290 kb)

Table S2 Sequences of the barcodes. (GIF 66 kb)

Table S3 Barcode combinations of the individuals. (DOCX 21 kb)

Fig. S1 Amplification gel pictures of locus 23 in 442 individuals. (DOCX 14 kb)

High Resolution Image (DOCX 19 kb)

Table S4 Statistics of genotype/phenotype correlation analysis on the basis of MLM. (TIFF 2584 kb)

Fig. S2 DNA sequencing map of sites in Nos. 1, 13, and 18 genes. (a) DNA sequencing map of site in the No. 1 gene. (b) DNA sequencing map of site in the No. 13 gene. (c) DNA sequencing map of site in the No. 18 gene. SNP site is indicated by black arrow. (XLSX 39 kb)

High Resolution Image (TIFF 11451 kb)
