# Supplementary figures and images for: Selection of growth-related genes and dominant genotypes in transgenic Yellow River carp Cyprinus carpio L
Source: Funct Integr Genomics. 2018 Apr 5;18(4):425–37. doi: 10.1007/s10142-018-0597-9 (PMC6004361; doi:10.1007/s10142-018-0597-9)

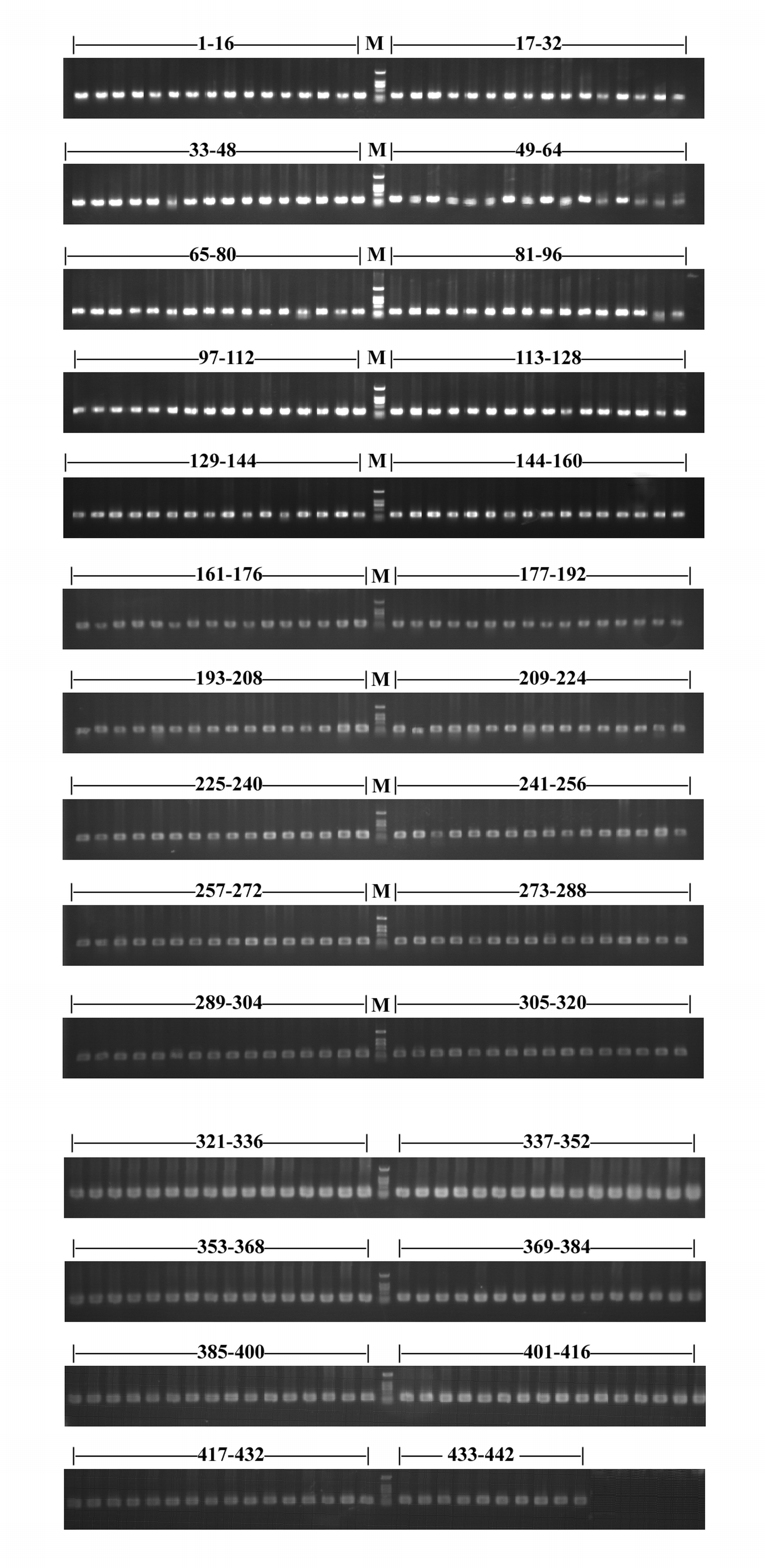

Supplement: Supplementary file 1 — Specific primers for each SNP site. (GIF 2290 kb) [file 10142_2018_597_Fig8_ESM.gif]

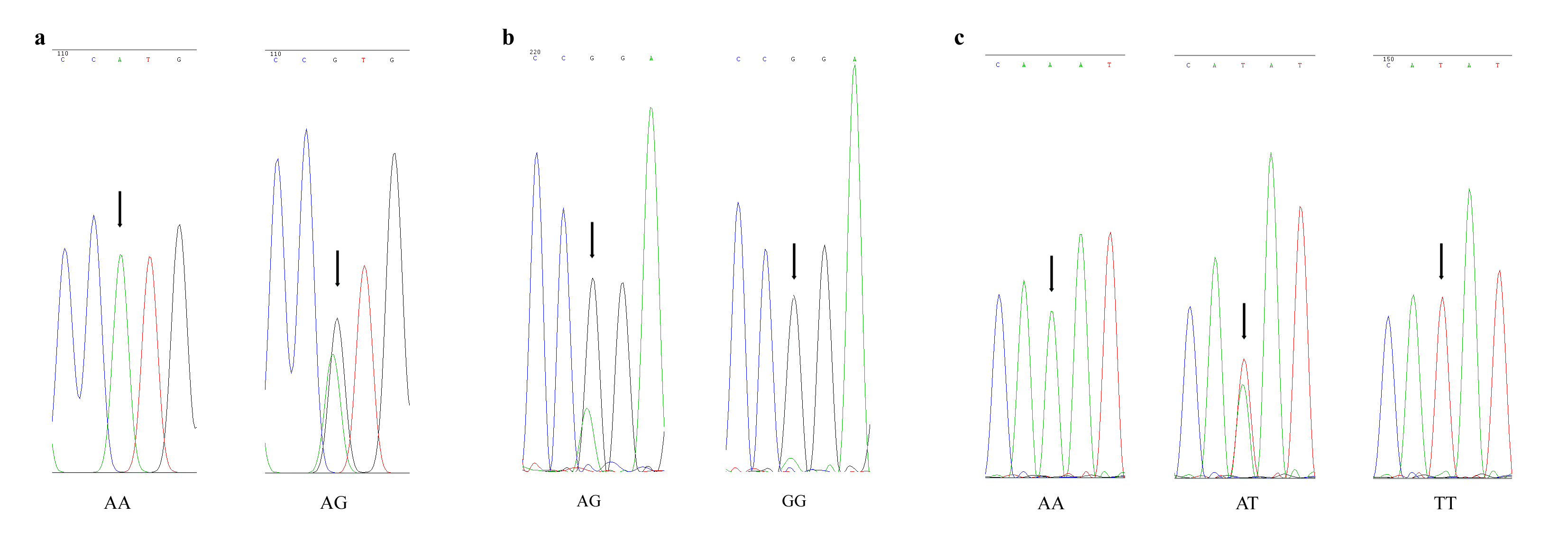

Supplement: Supplementary file 2 — Sequences of the barcodes. (GIF 66 kb) [file 10142_2018_597_Fig9_ESM.gif]

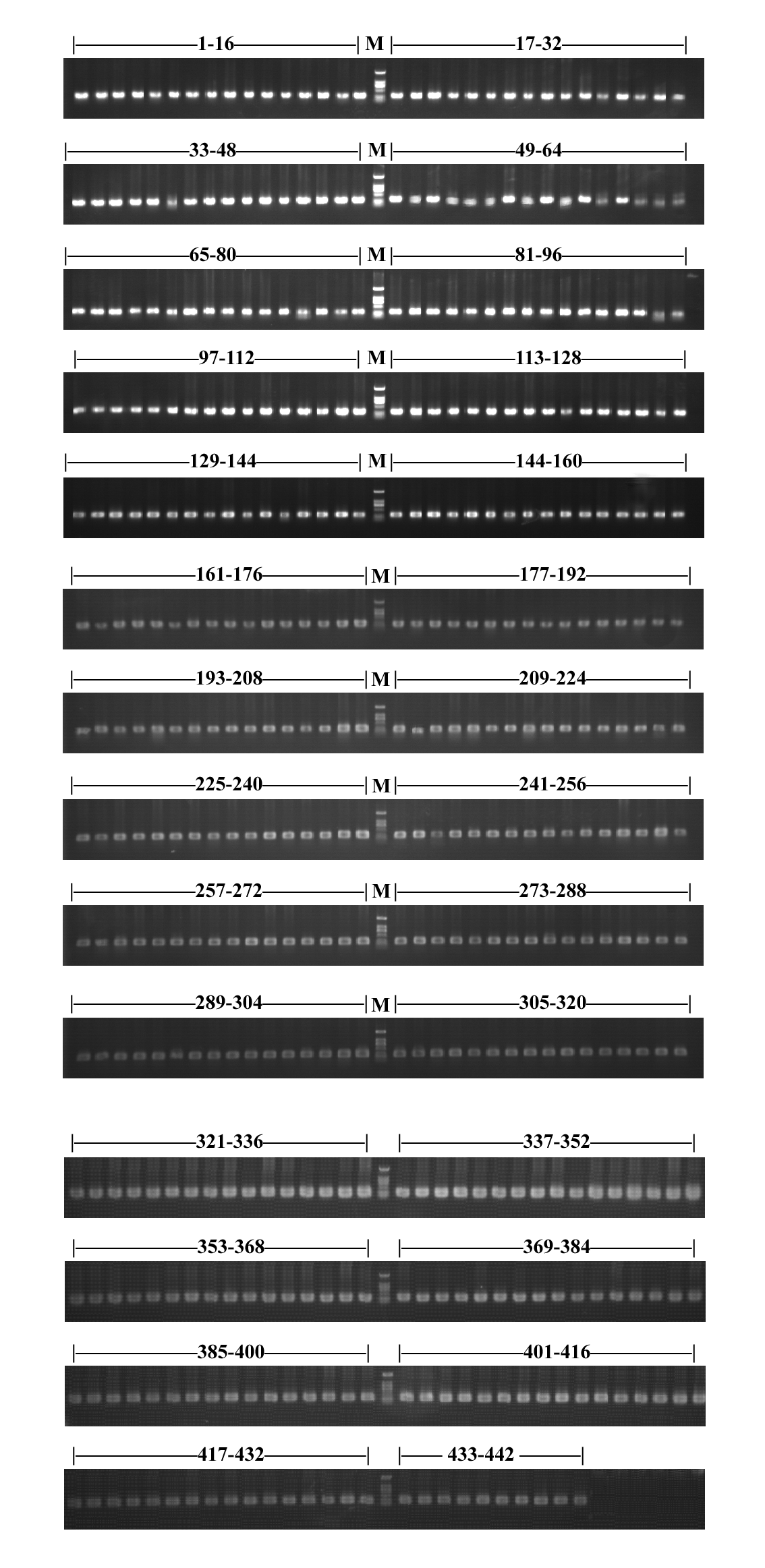

Supplement: Supplementary file 6 — Statistics of genotype/phenotype correlation analysis on the basis of MLM. (TIFF 2584 kb) [file 10142_2018_597_MOESM4_ESM.tif]

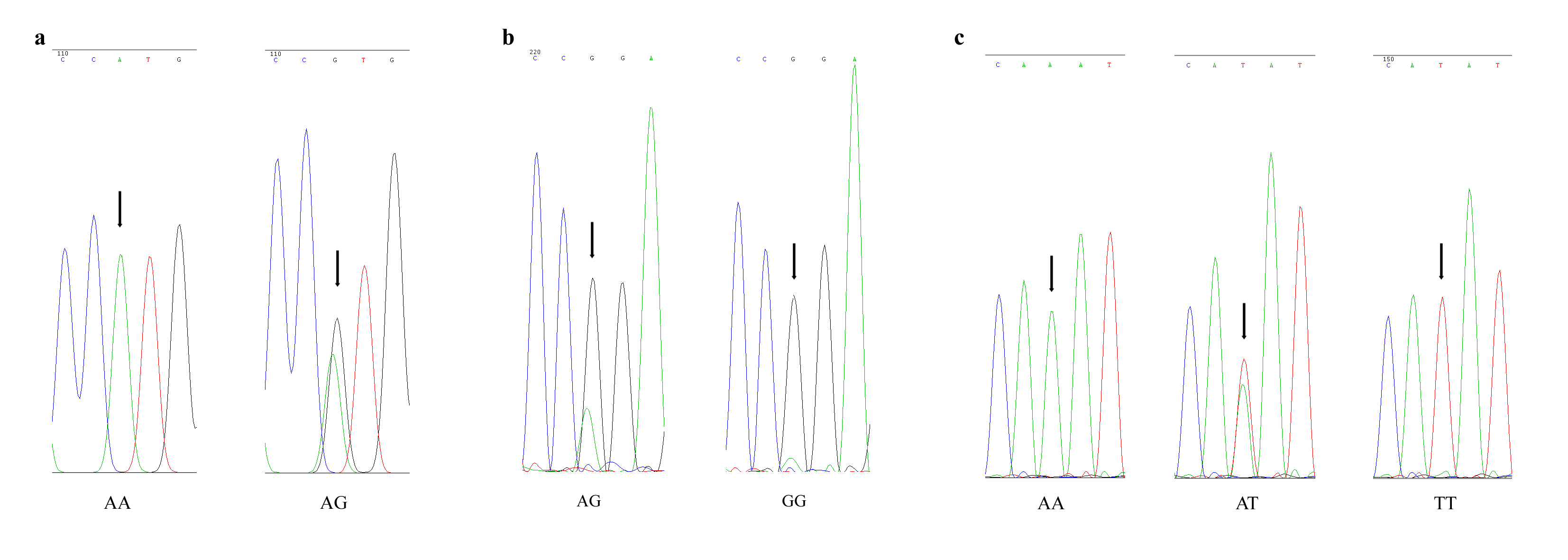

Supplement: Supplementary file 8 — High Resolution Image (TIFF 11451 kb) [file 10142_2018_597_MOESM6_ESM.tif]
