# Supplementary material for: Selection of growth-related genes and dominant genotypes in transgenic Yellow River carp Cyprinus carpio L
Source: Funct Integr Genomics. 2018 Apr 5;18(4):425–37. doi: 10.1007/s10142-018-0597-9 (PMC6004361; doi:10.1007/s10142-018-0597-9)
Supplement: Supplementary file 3 — Barcode combinations of the individuals. (DOCX 21 kb) [file 10142_2018_597_MOESM1_ESM.docx]

| primer | Sequences (5’ to 3’) | Usage |
| --- | --- | --- |
| 2LG45-1f | TTCAGAAAAAACTAAACAAA | locus1 cloning |
| 2LG45-1r | AGCAAGAAACAGACCAAC |  |
| 3LG42-1-1f | TCTTCTAAAATAAAATGAATGT | locus2 cloning |
| 3LG42-1-1r | ATTTCTCTATCTCCAGTGTCT |  |
| 4LG38-2-1f | ATGTGTTGGAAAGACGAC | locus3 cloning |
| 4LG38-2-1r | ATGTTAAAGTTAGTTCAGGAT |  |
| 4LG38-3-1f | GTTGGTGTTTCTTGGAG | locus4 cloning |
| 4LG38-3-1r | GTCATTTTAATAAAGTGTAATAC |  |
| 5LG13-1-1f | GTTCTATCAAAAAGTCCTTC | locus5 cloning |
| 5LG13-1-1r | AACTGAGCTATCGGGG |  |
| 5LG13-2-1f | CTAAACCCTTGATCTGTATC | locus6 cloning |
| 5LG13-2-1r | AGCATGTAAAACACAAGAGT |  |
| 6LG8-1f | TTAAAAAGGCGGTTCAT | locus7 cloning |
| 6LG8-1r | AGGTAAAACAAAATCCAAA |  |
| 7LG33-1-3f | ACCTGGCAACCCTGAT | locus8 cloning |
| 7LG33-1-3r | TCCAAACAAATTTTTCTTCT |  |
| 7LG33-2-1f | CAAGTCGCTGTGCCGT | locus9 cloning |
| 7LG33-2-1r | CCATTCCCAAACTCAAGC |  |
| 8LG3-2f | ACATGATGGCATCAAGC | locus10 cloning |
| 8LG3-2r | TGTACCCATCCTCTGATG |  |
| 9LG6-1-2f | CCCTTATTTCTTTTATGATG | locus11 cloning |
| 9LG6-1-2r | ACGCCATCTGCTCAAT |  |
| 9LG6-2-1f | ATGAGAACTACAACTTCCG | locus12 cloning |
| 9LG6-2-1r | TTGTGGTGAAAGAAGGTT |  |
| 10LG6-1-1f | CCCAGGACTCTGTGGTT | locus13 cloning |
| 10LG6-1-1r | CTAAAATGAACTACGAGAAGC |  |
| 10LG6-2-2f | TGTCATTTCCCCCTCTC | locus14 cloning |
| 10LG6-2-2r | TCTTGTGTAGGTGATACTGAG |  |
| 11LG11-1f | ACCGTCGAAGAGAAGCA | locus15 cloning |
| 11LG11-1r | GTCCGCCGGGGATCTC |  |
| 12LG40-3f | TTCTCACTTTTCTCTTCTGC | locus16 cloning |
| 12LG40-3r | GATATCATAAAGCAAGTTTTG |  |
| 13C316-1f | AGGCTCTTGATTGGTGTC | locus17 cloning |
| 13C316-1r | TTGGCATCAGCTATTCAT |  |
| 14C626-1f | GGACCTTCATGTGAACTTT | locus18 cloning |
| 14C626-1r | CCGTCAGTGTGCAAAGA |  |
| 15C1040-1-1f | CACTGCGACTCACAAGAC | locus19 cloning |
| 15C1040-1-1r | TCCCTGATCTGAAGAGTTT |  |
| 15C1040-2-1f | TACCATATAAAGTGAAAACATT | locus20 cloning |
| 15C1040-2-1r | TGTTTCATTTGACAAGGC |  |
| 16C1094-2f | GAGGAGCCCAAACTGT | locus21 cloning |
| 16C1094-2r | TTAGGTGCCACAGATTAA |  |
| 17C1097-1-1f | TTTCAAGTAGGATGGGTG | locus22 cloning |
| 17C1097-1-1r | GGAGAGTAAAGACATTTAAGTT |  |
| 17C1097-2-1f | GGACAGGTAAAAAAGCCT | locus23 cloning |
| 17C1097-2-1r | ATCTGGAGGGTTGTTGG |  |
| 18C1169-1-2f | AATGAGCTAAAAGCCCC | locus24 cloning |
| 18C1169-1-2r | TTTCACACACGCACACA |  |
| 18C1169-2-1f | ATAGGGTGAAGGTGACG | locus25 cloning |
| 18C1169-2-1r | GTACATCTCTGCTCTCCTCT |  |
| 19C10792-3f | GTTAGGGGAATGAAACG | locus26 cloning |
| 19C10792-3r | GTCATTGGAACATGATTCA |  |
| 20C2940-1f | GATGGCGGAGTACCTG | locus27 cloning |
| 20C2940-1r | AATGTTTATTCATCAAATTCA |  |
| 21C653-1-1f | AAAATGGTCGCAGTCTG | locus28 cloning |
| 21C653-1-1r | AAAGATGAAGTTGCAGAAT |  |
| 21C653-2-1f | AGGAAAAAATCTGTGGG | locus29 cloning |
| 21C653-2-1r | TCCTCATTAGTGTCATTACC |  |
| 22C620-1-1f | TTCATATGTATTTGTGTACAAA | locus30 cloning |
| 22C620-1-1r | TCGGTTTTTAATTCGG |  |
| 22C620-2-1f | GTGTTTCCTAGTTCTATGGTAT | locus31 cloning |
| 22C620-2-1r | CTGAAGACACTTACAGAACCT |  |
| 23C11016-1-1f | TATGACAAAGGTAAAGCATACA | locus32 cloning |
| 23C11016-1-1r | AAACTGCGCATGCTCC |  |
| 23C11016-2-2f | CGAGACTCATGTCGACC | locus33 cloning |
| 23C11016-2-2r | CGCAGGGGTATTACTGT |  |
| 24C28863-1-4f | GAGCTGATAAGCTGGTG | locus34 cloning |
| 24C28863-1-4r | GGACATCATGTACAAAGTTA |  |
| 24C28863-2-1f | GGCTGAACTTTACTGTGG | locus35 cloning |
| 24C28863-2-1r | GTTCACAAAGAAATATGCAG |  |
