# Supplementary material for: Selection of growth-related genes and dominant genotypes in transgenic Yellow River carp Cyprinus carpio L
Source: Funct Integr Genomics. 2018 Apr 5;18(4):425–37. doi: 10.1007/s10142-018-0597-9 (PMC6004361; doi:10.1007/s10142-018-0597-9)
Supplement: Supplementary file 4 — Amplification gel pictures of locus 23 in 442 individuals. (DOCX 14 kb) [file 10142_2018_597_MOESM2_ESM.docx]

| Barcode | Sequences (5’ to 3’) | Barcode | Sequences (5’ to 3’) |
| --- | --- | --- | --- |
| F1 | ATACAGCTGTACTGACGAGC | R3 | TGTAGTGTAGTAGCGTCGAC |
| F2 | TCATCTGAGCGTGCTATCGT | R4 | TCGTGTGAGTACTAGTCTGC |
| F3 | CTGCGAGTGAGATCAGCATA | R5 | CTCGTCTACGACTGTAGAGT |
| F4 | ACGTGATGCTCATATCGAGC | R6 | TACTGAGACTCGACTGAGTC |
| F5 | TACTGTGACGCAGACACTGT | R7 | TCATGCATGCACGCTACATG |
| F6 | CACACTGTCAGATGAGTGAC | R8 | TCAGACTAGTCGTGCATGAG |
| F7 | AGCGATGATGCGATCGAGTA | R9 | GAGATCGACTATCTGCTCAC |
| F8 | AGATAGACAGAGCTGCTCAC | R10 | GCGCGATATAGAGACTACGA |
| F9 | CTAGTGCTGTCACATAGCGT | R11 | AGTGTCGTATACGTGACGTG |
| F10 | AGATGCTACGCTGAGAGACT | R12 | TGTCGTACTCATACTCAGCG |
| F11 | TCATCTCTCTGCGATGCAGA | R13 | CTGCTCAGAGTCTGAGATCT |
| F12 | CTGATGAGCATGCAGTAGCA | R14 | GCACAGATGAGACTGTAGCA |
| F13 | CAGTAGTACGACACATGCTG | R15 | CGTAGCTCTCAGCTAGTCTA |
| F14 | GCGTCTGTCATGTCTGACAT | R16 | AGTGTCAGTCGTGTCTCAGA |
| F15 | TGCGCGTACTCTAGTACAGA | R17 | TAGCATGCGTACGAGAGACT |
| F16 | TGACAGAGAGAGAGATGCGT | R18 | GTATCAGCGTGTACGTCTAG |
| F17 | GAGCAGTCTAGTAGACGTCA | R19 | GCTATCGCATCTGTCATCGA |
| F18 | GATCGCGTACAGCACTATCA | R20 | CATCATCACGCTGTGTGTAC |
| F19 | ACGTCTACAGAGACTGTGTG | R21 | CGACACACTATCGACGATGT |
| F20 | ACTCAGTCTGAGAGCTATGC | R22 | ATAGACGTACGTATCAGCGC |
|  |  | R23 | GTGACGAGTCACTATCATCG |
| R1 | ACACAGCACAGTCATCGCTA | R24 | GAGTACACACATACGAGCAG |
| R2 | AGTCGCACACTGTCATCTCT | R25 | TCACTGCATACGCACTACAG |
