# Supplementary material for: Selection of growth-related genes and dominant genotypes in transgenic Yellow River carp Cyprinus carpio L
Source: Funct Integr Genomics. 2018 Apr 5;18(4):425–37. doi: 10.1007/s10142-018-0597-9 (PMC6004361; doi:10.1007/s10142-018-0597-9)
Supplement: Supplementary file 5 — High Resolution Image (DOCX 19 kb) [file 10142_2018_597_MOESM3_ESM.docx]

|  | F1 | F2 | F3 | F4 | F5 | F6 | F7 | F8 | F9 | F10 | F11 | F12 | F13 | F14 | F15 | F16 | F17 | F18 | F19 | F20 |
| --- | --- | --- | --- | --- | --- | --- | --- | --- | --- | --- | --- | --- | --- | --- | --- | --- | --- | --- | --- | --- |
| R1 | 1 | 26 | 51 | 76 | 101 | 126 | 151 | 176 | 201 | 226 | 251 | 276 | 301 | 326 | 351 | 376 | 401 | 426 | 451 | 476 |
| R2 | 2 | 27 | 52 | 77 | 102 | 127 | — | 177 | — | 227 | 252 | 277 | 302 | 327 | 352 | 377 | 402 | 427 | 452 | 477 |
| R3 | 3 | 28 | 53 | 78 | 103 | — | — | 178 | — | 228 | 253 | 278 | 303 | 328 | 353 | 378 | 403 | 428 | 453 | — |
| R4 | — | 29 | 54 | 79 | 104 | 129 | — | 179 | — | 229 | 254 | 279 | 304 | 329 | 354 | 379 | 404 | 429 | 454 | — |
| R5 | 5 | 30 | 55 | 80 | 105 | 130 | — | 180 | — | 230 | 255 | 280 | 305 | 330 | 355 | 380 | 405 | 430 | 455 | — |
| R6 | 6 | 31 | 56 | 81 | 106 | — | — | 181 | — | 231 | 256 | 281 | 306 | 331 | 356 | 381 | 406 | 431 | 456 | 481 |
| R7 | 7 | 32 | 57 | 82 | 107 | 132 | — | 182 | — | 232 | 257 | 282 | 307 | 332 | 357 | 382 | 407 | — | — | 482 |
| R8 | — | 33 | 58 | 83 | 108 | 133 | — | 183 | — | 233 | 258 | 283 | 308 | 333 | 358 | 383 | 408 | — | 458 | 483 |
| R9 | 9 | 34 | 59 | 84 | 109 | 134 | — | — | — | 234 | 259 | 284 | 309 | 334 | 359 | 384 | — | 434 | 459 | 484 |
| R10 | 10 | 35 | — | 85 | 110 | 135 | — | — | 210 | 235 | 260 | 285 | 310 | 335 | 360 | — | 410 | 435 | 460 | 485 |
| R11 | 11 | 36 | 61 | 86 | 111 | 136 | — | 186 | 211 | 236 | 261 | 286 | 311 | 336 | 361 | 386 | 411 | 436 | 461 | 486 |
| R12 | 12 | 37 | 62 | 87 | 112 | 137 | — | 187 | — | 237 | 262 | 287 | 312 | 337 | 362 | 387 | 412 | 437 | 462 | 487 |
| R13 | 13 | 38 | 63 | 88 | 113 | 138 | 163 | 188 | 213 | 238 | 263 | 288 | 313 | 338 | 363 | 388 | 413 | 438 | 463 | 488 |
| R14 | 14 | 39 | 64 | 89 | — | — | 164 | 189 | 214 | 239 | 264 | 289 | 314 | 339 | 364 | 389 | 414 | 439 | 464 | 489 |
| R15 | 15 | 40 | 65 | 90 | 115 | 140 | 165 | 190 | — | 240 | 265 | 290 | 315 | 340 | 365 | 390 | 415 | 440 | 465 | — |
| R16 | 16 | 41 | 66 | 91 | 116 | 141 | 166 | 191 | — | 241 | 266 | 291 | 316 | 341 | 366 | 391 | 416 | 441 | 466 | — |
| R17 | 17 | 42 | 67 | 92 | 117 | 142 | 167 | 192 | — | 242 | 267 | 292 | 317 | 342 | 367 | 392 | 417 | 442 | — | — |
| R18 | 18 | 43 | 68 | 93 | 118 | 143 | 168 | 193 | 218 | 243 | 268 | 293 | 318 | 343 | 368 | 393 | 418 | 443 | — | — |
| R19 | 19 | 44 | 69 | 94 | 119 | 144 | 169 | 194 | 219 | 244 | 269 | 294 | 319 | 344 | 369 | 394 | 419 | 444 | — | — |
| R20 | 20 | 45 | 70 | 95 | 120 | 145 | 170 | 195 | 220 | 245 | 270 | 295 | 320 | 345 | 370 | 395 | 420 | — | 470 | — |
| R21 | 21 | 46 | 71 | 96 | 121 | 146 | 171 | 196 | 221 | 246 | 271 | 296 | 321 | 346 | 371 | 396 | 421 | 446 | 471 | — |
| R22 | 22 | 47 | 72 | 97 | 122 | — | 172 | 197 | 222 | 247 | 272 | 297 | 322 | 347 | 372 | 397 | 422 | 447 | — | — |
| R23 | 23 | 48 | 73 | 98 | 123 | 148 | 173 | 198 | 223 | 248 | 273 | 298 | 323 | 348 | — | 398 | 423 | 448 | 473 | — |
| R24 | 24 | 49 | 74 | 99 | 124 | 149 | 174 | 199 | 224 | 249 | 274 | 299 | 324 | 349 | 374 | 399 | 424 | 449 | 474 | — |
| R25 | 25 | 50 | 75 | 100 | 125 | 150 | 175 | 200 | 225 | 250 | 275 | 300 | 325 | 350 | 375 | 400 | 425 | 450 | 475 | — |
